# TOOme: A Novel Computational Framework to Infer Cancer Tissue-of-Origin by Integrating Both Gene Mutation and Expression

**DOI:** 10.3389/fbioe.2020.00394

**Published:** 2020-05-19

**Authors:** Binsheng He, Jidong Lang, Bo Wang, Xiaojun Liu, Qingqing Lu, Jianjun He, Wei Gao, Pingping Bing, Geng Tian, Jialiang Yang

**Affiliations:** ^1^Academician Workstation, Changsha Medical University, Changsha, China; ^2^Geneis Beijing Co., Ltd., Beijing, China; ^3^Fujian Provincial Cancer Hospital, Fuzhou, China

**Keywords:** tissue-of-origin, somatic mutation, gene expression, random forest, cross-validation

## Abstract

Metastatic cancers require further diagnosis to determine their primary tumor sites. However, the tissue-of-origin for around 5% tumors could not be identified by routine medical diagnosis according to a statistics in the United States. With the development of machine learning techniques and the accumulation of big cancer data from The Cancer Genome Atlas (TCGA) and Gene Expression Omnibus (GEO), it is now feasible to predict cancer tissue-of-origin by computational tools. Metastatic tumor inherits characteristics from its tissue-of-origin, and both gene expression profile and somatic mutation have tissue specificity. Thus, we developed a computational framework to infer tumor tissue-of-origin by integrating both gene mutation and expression (TOOme). Specifically, we first perform feature selection on both gene expressions and mutations by a random forest method. The selected features are then used to build up a multi-label classification model to infer cancer tissue-of-origin. We adopt a few popular multiple-label classification methods, which are compared by the 10-fold cross validation process. We applied TOOme to the TCGA data containing 7,008 non-metastatic samples across 20 solid tumors. Seventy four genes by gene expression profile and six genes by gene mutation are selected by the random forest process, which can be divided into two categories: (1) cancer type specific genes and (2) those expressed or mutated in several cancers with different levels of expression or mutation rates. Function analysis indicates that the selected genes are significantly enriched in gland development, urogenital system development, hormone metabolic process, thyroid hormone generation prostate hormone generation and so on. According to the multiple-label classification method, random forest performs the best with a 10-fold cross-validation prediction accuracy of 96%. We also use the 19 metastatic samples from TCGA and 256 cancer samples downloaded from GEO as independent testing data, for which TOOme achieves a prediction accuracy of 89%. The cross-validation validation accuracy is better than those using gene expression (i.e., 95%) and gene mutation (53%) alone. In conclusion, TOOme provides a quick yet accurate alternative to traditional medical methods in inferring cancer tissue-of-origin. In addition, the methods combining somatic mutation and gene expressions outperform those using gene expression or mutation alone.

## Introduction

Metastatic cancer is a common clinical challenge for limited evidence to determine its primary origin. Patients with carcinoma of unknown primary (CUP) account for about 5% of total cancer patients (Shaw et al., 2007). CUP are usually heterogeneous, and can lead to dilemmas in diagnosing and treatment since the original tumor site is unknown (Rizwan and Zulfiqar, 2010). Clinically, CUP patients are generally treated with non-selective empirical chemotherapy, which usually leads to low survival rates (Kurahashi et al., 2013). Thus, identifying cancer tissue-of-origin (TOO) is critical in improving the treatment of cancer patients and extending their surviving time (Hudis, 2007; Varadhachary et al., 2008; Hyphantis et al., 2013).

There are several ancillary examinations in CUP identification, among which immunohistochemistry (IHC) is an important one. However, this method relies on the experiences of pathologists and is labor-intensive. As a result, it is inaccurate in most of the times (Huebner et al., 2007; Voigt, 2008; Centeno et al., 2010; Kandalaft and Gown, 2015; Janick et al., 2018). Positron emission tomography (PET) and computed tomography (CT) are also commonly used in the identification of CUP (Fencl et al., 2007; Kwee et al., 2010; Fu et al., 2019). The detection rate of conventional radiological imaging on primary carcinoma reach 20–27%, and that of PET reach 24–40% (Ambrosini et al., 2006). The detection accuracy of PET/CT is awfully low that it rarely brings help to identify the primary origin. Obstacles in image technology cause much difficulty of effective use of relative Carcinoma image to help tracing cancer tissue origin.

Molecular profiling of tissue-specific genes is also being used in CUP work-up. Quantities of large-scale profiles of different tumors have been used for diagnose. Molecular profiling is as well as or better than IHC, in terms of poorly differentiated or undifferentiated tumors (Oien and Dennis, 2012). Therefore, making use of molecular profiling has become a popular way for diagnosis of unknown origin. Comprehensive molecular profiles displayed in The Cancer Genome Atlas (TCGA) including copy number variation, somatic mutation, gene expression, microRNA expression, DNA methylation, and protein expression, are used to identifying human tumor types (Li et al., 2017). By analysis of tumor types from data of methylation and copy number variation, tissue of origin and molecular classification can be revealed (Hoadley et al., 2014). The methylation profile of metastasis in a meningeal melanocytic tumor is similar to that of primary tumor, and it is suggest that particular copy number variations may be associated with metastatic behavior (Küsters-Vandevelde et al., 2017). Methylation and copy number variation are DNA-level molecular profiling, which brought great help to identify tumor origins.

The copy number profile and gain or loss in specific chromosome regions have been researched by hybridization and cytogenetic-based methods (Baudis, 2007; Beroukhim et al., 2007). An *IDH1* somatic mutation in genomic profiling was revealed to bring great benefit to the diagnosis of cholangiocarcinoma and trace the primary origin in a malignancy (Sheffield et al., 2016). Marquard et al. (2016) obtained classification accuracy of 69% and 85% on 6 and 10 primary sited with somatic mutation, respectively, based on PM and CN classifier (classifiers with both point mutations and copy number aberrations) with cross-validation. Mutation of tumor-specific enrichment in certain genes, has been utilized to infer tumor localization, and Dietlein and Eschner (2014) developed a tool with mutation spectra to infer cancer origins with a prediction specificity of 79% (Lawrence et al., 2014). As a DNA-level molecular profiling, SNP, that is somatic mutation, can be used as a very useful tool to infer the tissue of origins.

A lot of RNA-level gene expression profile have been explored to identify the cancer tissue of origin (Erlander et al., 2004; Qu et al., 2007; Gross-Goupil et al., 2012; Greco, 2013; Hainsworth et al., 2013). Erlander et al. (2011) have demonstrated that the gene expression value of samples detected in metastatic tumor is similar to that in the original tumor under condition of CUP. Centeno et al. (2010) developed a hybrid model by integrating expression profiling and IHC for microRNA-based qRT-PCR test on identification of cancer tissue origin, with 85% of the cases correctly identified (Rosenwald et al., 2010). Bloom et al. (2004) utilized artificial neural networks (ANNs) to predict the unknown cancer tissue origin with mean accuracy of 83–88% in different platforms.

Numerous researches have utilized molecular profiles, such as copy number variation, somatic mutation, gene expression, and so on for predicting cancer tissue origin. However, the accuracy of prediction was not satisfying. Identifying cancer tissue origin by combining somatic mutation and gene expression profiling on DNA level and RNA level, respectively, is first proposed in this study. Firstly, we obtained the data of somatic mutation and gene expression profiling from International Cancer Genome Consortium (ICGC) Database. Machine learning methods can help to improve the performance on prediction of cancer tissue origin. We aim to obtain better performance in predicting cancer tissue origin, by the combination of somatic mutation and gene expression profiling, based on random forest. Machine learning algorithm, such as logistic regression can be used to select gene (Kao et al., 2006). However, random forest algorithm (Sandri and Zuccolotto, 2006) was chosen as the gene selection algorithm in this study due to its advantage, good robustness and easy to use. Finally, we used random forest algorithm for classification of cancers. Experiment results showed that higher accuracy can be obtained by using the method proposed in this study.

## Materials and Methods

### Gene Expression Data

Gene expression profile was downloaded from ICGC Database version release-26^1^. Each gene is named by Gene Symbol ID. The value of gene expression in each labeled sample is normalized by TPM. After deduplication, samples were extracted for combination with SNP samples.

### Somatic Mutation Data

The somatic mutation data was downloaded from ICGC Database version release-28^2^. Each gene is named by Ensembl Gene ID. For Gene Symbol ID is most widely used in paper, the Ensembl Gene ID of gene name in somatic mutation data was converted to Gene Symbol ID. The samples are deduplicated according to information of ICGC-donor-ID, chromosome, and locus in chromosome and gene-affected. Each sample was labeled by its type of cancer.

### Data Combination

The gene expression and somatic mutation data were merged into one feature matrix. For labeled samples with gene expression array data only involves in 21 cancer types, and samples with Skin Cutaneous Melanoma (SKCM) were removed for it contributes to the major metastasis cancers. The sample with somatic mutation data whose label was not included in these 20 cancer types was removed. Then, the shared sample data was chosen, therefore the samples data after filtering is obtained from 20 different cancer types. An M^∗^N matrix was generated, where M and N represents the number of sample and gene, respectively.

### Gene Selection

Because gene sequencing and mutation detection are costly and time consuming, a scale reduction of gene number is necessary. There are many feature selection algorithms, like Lasso, PCA (Malhi and Gao, 2005; Muthukrishnan and Rohini, 2016) and etc. The Random forest (Breiman, 2001; Sandri and Zuccolotto, 2006) was a supervised learning algorithm, which is an ensemble learning algorithm based on decision tree and was used to select genes. Best performance was obtained by using 80 selected genes. $\sqrt{n}$ genes were used in a tree, where n represents the number of genes. At the process of splitting node, Gini index was used, which is calculated by formula:

(1) G⁢i⁢n⁢i⁢(p)=∑k=1Kpk⁢(1-pk)=1-∑k=1Kpk2

Where *p* represents the weight referring to frequencies of cancers in a node, *k* represents the number of cancers and *p*_*k*_ represents the weight of the *k*th cancer. The variable importance measures of *i*th gene in node *m*, that is the Gini index variation after splitting of node *m*, is calculated by formula:

(2) $$V\,I\,M_{i\,m}^{\left(G\,i\,n\,i\right)}=G\,I_{m}-G\,I_{l}-G\,I_{r}$$

Where *m* is a node in *M*, which is a set of nodes, $V\,I\,M_{i\,m}^{\left(G\,i\,n\,i\right)}$ represents variable importance measures of *i*th gene in node *m*, the *GI*_*m*_ represents the Gini index before splitting, *GI*_*l*_ and *GI*_*r*_ represents the Gini index of two new node after splitting, respectively. The importance of the *i*th gene, in the *t*th tree is calculated by formula:

(3) $$V\,I\,M_{t\,i}^{\left(G\,i\,n\,i\right)}=\sum_{m\in M}V\,I\,M_{i\,m}^{\left(G\,i\,n\,i\right)}$$

Where $V\,I\,M_{t\,i}^{\left(G\,i\,n\,i\right)}$ represents the importance of the *i*th gene in the *t*th tree. If the set of trees is *T*, the importance of the *i*th gene in all the tree is calculated by formula:

(4) $$V\,I\,M_{i}^{\left(G\,i\,n\,i\right)}=\sum_{t=1}^{T}V\,I\,M_{t\,i}^{\left(G\,i\,n\,i\right)}$$

Where $V\,I\,M_{i}^{\left(G\,i\,n\,i\right)}$ is the importance of the *i*th gene in all trees. We sorted the importance scores of all genes, then the top *H* genes were selected, where *H* is the variable number of genes that can be set to find the best result.

### Multi-Classifier Random Forest

The random forest is actually a special method of bagging that using the decision tree as a model in bagging (Breiman, 2001; Meyer et al., 2019). First, the bootstrap method is used to generate *m* training sets, which is a set of samples. Then, each training set is used to construct a tree. $\sqrt{n}$ genes are used in a tree, where n represents the number of selected genes. When splitting a node, not all the genes are used to optimize the metric Gini index used in this study, a part of genes is randomly extracted instead. An optimal solution can be found among the extracted genes, and applied to node splitting. Leaf node in the tree records which gene is used to determine the cancer type, and each leaf node represents the last judged cancer type. The predicted cancer type is given by maximum votes from decision tree.

### Statistical Analysis

The metric of precision, recall and F1 score were used to evaluate the performance of the model. True-positive, false-positive, true-negative and false-negative are abbreviated as TP, FP, TN, and FN, respectively. Precision is calculated by (*TP*)/(*TP* + *FP*), which indicates the ability of classifier to differentiate positive from negative cases. Recall is calculated by (*TP*)/(*TP* + *FN*), which indicates the ability of classifier to recognize all positive cases. The *F*1 score is calculated by (2*recall*precision)/(*recall* + *precision*). Each individual cancer type is calculated by these metrics, and the cohort metric adopt the mean report. The entire cohort is calculated by accuracy, reported as (*TP* + *TN*)/(*totalcases*). Ten times 10-fold cross validation is used to obtain the metric report, whose average is treated as the result metric.

### Gene Annotation

The functions annotation of specific gene set was given. Geno ontology (Ye et al., 2006; Waardenberg et al., 2016) was used as enrichment analysis database. Gene clustering and visualization was realized by R package cluaterProfiler and gogadget (Yu et al., 2012; Nota, 2016).

## Results

### The Workflow of TOOme

**FIGURE 1:**
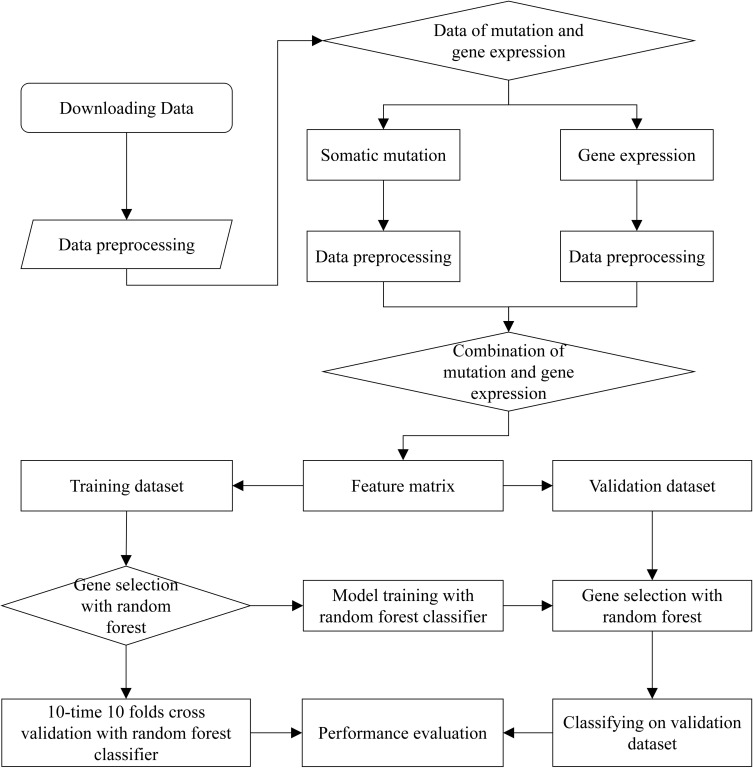
The complete workflow of prediction on cancer tissue origin.

The complete workflow of prediction on cancer tissue origin is shown in Figure 1. The process can be split into three steps. At the first step, we download the raw data from ICGC Database, and extracted the effective information to obtain preliminary data of somatic mutation and gene expression profiling. At the second step, we filtered the data of somatic mutation and gene expression profiling, respectively. Then, samples with both somatic mutation data and gene expression proofing were used to form feature matrix. As a result, the generated feature matrix was used for gene selection. At the third step, most of the samples were utilized to train the model with 10-time 10 folds cross validation by using random forest classification algorithm. We carried out numerous experiments to evaluate the performance of the proposed method.

### Data Used in This Study

We used ICGC version 26 and 28 databases, with Gene expression profile and somatic mutation information to classify tumor samples. The allele mutation in somatic mutation data can be A/G, C/T, C/A, and etc. For it is hard to distinguish mutation types with limited relative information and tools, we consider all kinds of allele mutation as gene mutation and count the number of gene mutation of each sample. Different from somatic mutation data, gene expression profile array data is directly used. The sample distribution of each cancer is showed in Table 1, where samples suffer from BRCA are much more than from other cancers. Considerable prediction results can be obtained by our model. The precision, recall and *F*_1_ score, showed in Table 2, reach 99.86%, 99.47% and 99.67%, respectively.

**TABLE 1:** Sample distribution of each cancer from ICGC database.

**Available cancer types**	**Abbreviation**	**Samples**	
		**Amount**	**Percentage**
Bladder urothelial carcinoma	BLCA	294	4.20%
Breast invasive carcinoma	BRCA	970	13.84%
Cervical squamous cell carcinoma and endocervical adenocarcinoma	CESC	241	3.44%
Colon adenocarcinoma	COAD	390	5.57%
Glioblastoma multiforme	GBM	148	2.11%
Head and neck squamous cell carcinoma	HNSC	460	6.56%
Kidney renal clear cell carcinoma	KIRC	345	4.92%
Kidney renal papillary cell carcinoma	KIRP	216	3.08%
Acute myeloid leukemia	LAML	121	1.73%
Brain lower grade glioma	LGG	433	6.18%
Liver hepatocellular carcinoma	LIHC	282	4.02%
Lung adenocarcinoma	LUAD	475	6.78%
Lung squamous cell carcinoma	LUSC	411	5.87%
Ovarian serous cystadenocarcinoma	OV	185	2.64%
Pancreatic adenocarcinoma	PAAD	134	1.91%
Prostate adenocarcinoma	PRAD	374	5.34%
Rectum adenocarcinoma	READ	137	1.95%
Stomach adenocarcinoma	STAD	412	5.88%
Thyroid carcinoma	THCA	486	6.93%
Uterine corpus endometrial carcinoma	UCEC	494	7.05%
Total		7008	100%

**TABLE 2:** Performance of classification of combination of somatic mutation and gene expression by using 80 genes.

**Cancer type**	**Precision**	**Recall**	**F1-score**	**Support**	**Specificity**
BLCA	0.8906	0.9354	0.9124	294.0000	0.9950
BRCA	0.9987	0.9947	0.9967	970.0000	0.9998
CESC	0.9148	0.8859	0.9001	241.0000	0.9971
COAD	0.7548	0.9644	0.8468	390.0000	0.9815
GBM	0.9940	1.0000	0.9970	148.0000	0.9999
HNSC	0.9916	1.0000	0.9958	460.0000	0.9994
KIRC	0.9850	0.9516	0.9680	345.0000	0.9992
KIRP	0.9344	0.9630	0.9485	216.0000	0.9979
LAML	1.0000	1.0000	1.0000	121.0000	1.0000
LGG	0.9926	0.9977	0.9952	433.0000	0.9995
LIHC	0.9925	0.9844	0.9884	282.0000	0.9997
LUAD	0.9358	0.9448	0.9403	475.0000	0.9953
LUSC	0.9408	0.9000	0.9199	411.0000	0.9965
OV	1.0000	0.9946	0.9973	185.0000	1.0000
PAAD	0.9378	0.9552	0.9464	134.0000	0.9988
PRAD	0.9973	1.0000	0.9987	374.0000	0.9998
READ	0.7569	0.1591	0.2627	137.0000	0.9990
STAD	0.9947	0.9976	0.9961	412.0000	0.9997
THCA	1.0000	0.9979	0.9990	486.0000	1.0000
UCEC	0.9673	0.9816	0.9744	494.0000	0.9975
Accuracy	0.9577	0.9577	0.9577	0.0000	

In this study, there are 371 samples with metastasis, where 352 samples are SKCM. To avoid unbalanced distribution of samples, we removed all the SKCM samples with metastasis. Only 19 samples with metastasis were used as test dataset.

### Performance Evaluation

**FIGURE 2:**
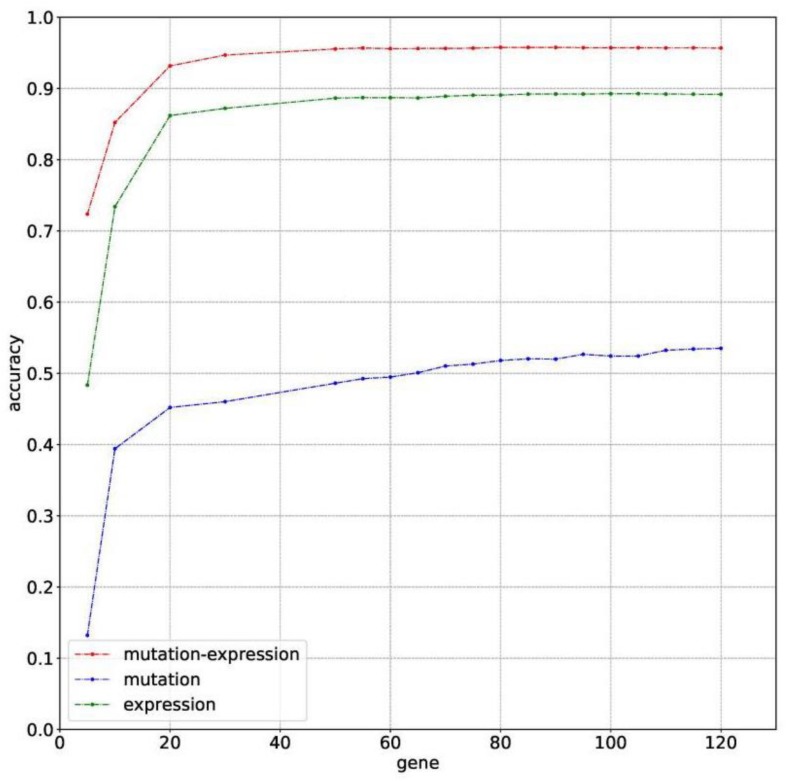
The classification accuracy of using somatic mutation, gene expression and combination of somatic mutation and gene expression, respectively.

The classification accuracies obtained by using data of somatic mutation, gene expression profiling and both of them, under condition of using different number of genes, have been compared in Figure 2. Motivated by Ma et al. (2006) that five genes can be used to solve a 32-type classification problem, five was chosen as the minimum number of genes. For gene sequencing and mutation detection are costly and time consuming, 120 was chosen as the maximum number of genes. A lot of experiments have been done using the prepared data between the interval from 5 to 120. For using small number of genes did not obtain satisfying classification performance, the interval between number of genes was set to 10 or even larger until the number of genes equals to 50. Then the interval was set to 5 for fine tuning, based on small fluctuation by changed number of genes.

Results with 10-time 10 folds cross validation on training dataset are shown in Figure 2 that accuracy of using data of both somatic mutation and gene expression profiling is always higher than that of only using one of it. The best result of them are 95.77%, 53.51%, and 89.28%, obtained by using 80, 120, and 105 genes, respectively. Results shows that gene expression can make much contribution to obtain higher accuracy than data of somatic mutation. However, a combination of them achieved best classification performance.

As for the test dataset, we conducted experiments by using the chosen 80 genes in training model. The overall classification accuracy is 89.47%. Table 3 shows the prediction probabilities of each sample on each cancer. The value on the table highlighted by color of green, yellow, and pink presents high, middle, and low probabilities, respectively, of predicting a sample to a cancer type. We obtained considerable prediction accuracy on sample with BRCA and THCA. Each sample was correctly predicted to the same as the true label. A sample whose true label is CESC was predicted to UCEC. A sample whose true label is BRCA was predicted to LGG with a terrible probability 1.65%. In this condition, we considered that little error on classification is tolerable.

**TABLE 3:** Prediction probabilities of each samples on each cancer.

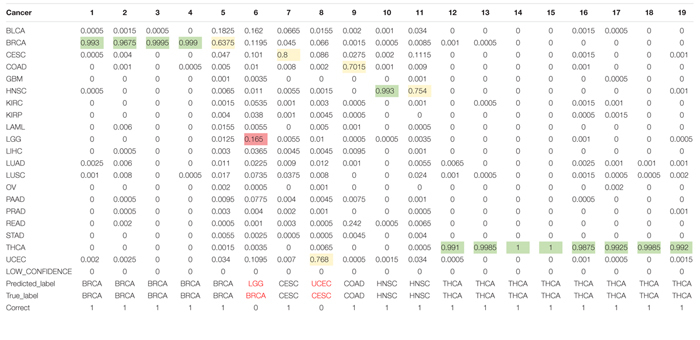

### Mean Value of Gene Expression and Somatic Mutations on Each Cancer

**FIGURE 3:**
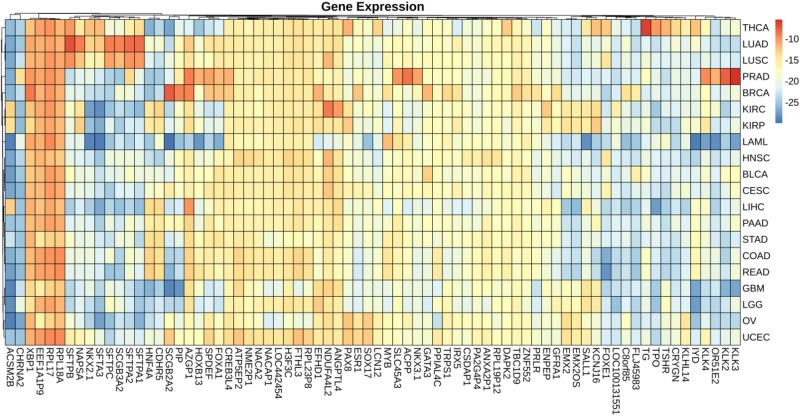
Heatmap of mean value of gene expression on each cancer.

**FIGURE 4:**
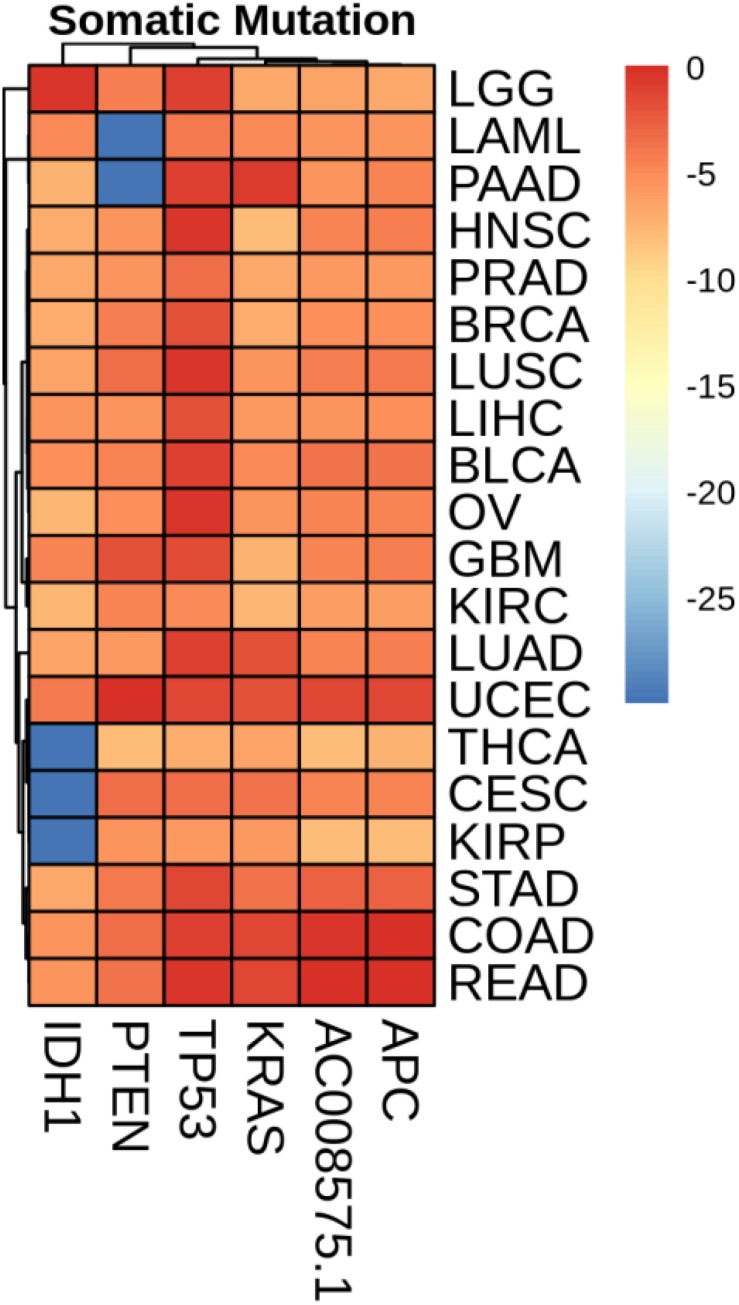
Heatmap of mean value of somatic mutations on each cancer.

We plotted the heatmap of mean value of gene expression and somatic mutations on each cancer. In Figure 3, the rows represent 74 genes of gene expression and columns denote the cancers. In Figure 4, the rows represent six genes of somatic mutation and columns represent the cancers. The mean value of gene expression and somatic mutation on a logarithmic scale was plotted with relative color. A color bar was used to display the value difference. Cancers that fell into cluster at horizontal axis had a similar value between gene expression or mutation number. The genes were also clustered at vertical axis based on the similarity between cancers.

## Discussion

Data of somatic mutation and gene expression profiling can be used to identify the primary site of tumors. However, it was the first time to identify the cancer tissue origin by using both data of somatic mutation and gene expression profiling. We carried out experiments by using 7008 samples with combination of data of somatic and gene expression profiling among 20 cancers. By comparing the performance of them, we obtained highest accuracy by leveraging both of the data of somatic mutation and gene expression profiling.

The primary analysis tool we used was random forest (Breiman, 2001; Sandri and Zuccolotto, 2006), a machine learning algorithm that can be used for gene selection and tumor classification. We chose top-rank 80 genes, where 6 genes and 74 genes are corresponding to mutation and expression, respectively, for classification. Therefore, it showed that data of somatic mutation performs worse than gene expression profiling on prediction of cancer tissue origin. Our method obtained 96% overall accuracy on the training dataset. The performance is maintained considerably on the external cohorts, and the overall accuracy on sample with metastatic disease is 89%. Our model cannot provide good performance on physiologically proximal cancers, such as uterine corpus endometrial carcinoma and cervical squamous cell carcinoma and endocervical adenocarcinoma. The endometrial and ovarian endometrioid carcinomas evolve from similar precursor endometrial epithelial cells; many researches are involved in the molecular pathogenesis of the endometrial and ovarian endometrioid carcinomas (McConechy et al., 2014).

**FIGURE 5:**
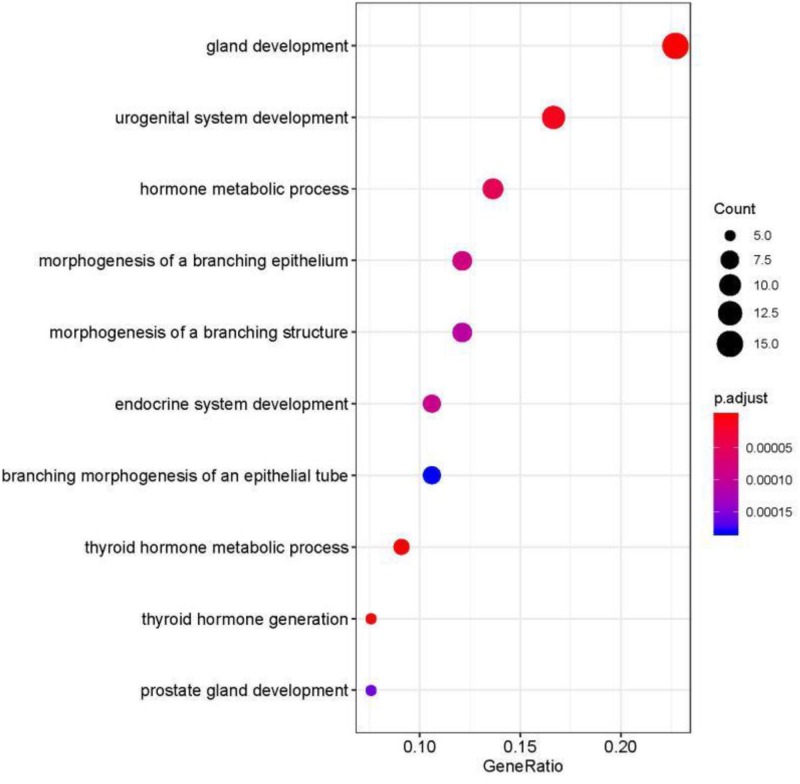
Selected top-rank 80 genes enriched in cellular component, biological process and molecular function.

We studied the role that gene plays in cellular component, biological process and molecular function. Figure 5 shows the top-rank 80 genes selected by random forest algorithm. The selected genes were enriched in hormone metabolic process, tissue and organ development and hormone-mediated signaling pathway, specifically in gland development, urogenital system development, hormone metabolic process, morphogenesis of a branching epithelium, morphogenesis of a branching structure, endocrine system development, branching morphogenesis of an epithelial tube, thyroid hormone metabolic process, thyroid hormone generation and prostate gland development. For example, *APC* plays a significant role in discovering pathogenesis of soft tissue tumors (Kuhnen et al., 2000). Birnbaum et al. (2012) investigated what role the *APC* gene play in colorectal cancer, at the investigation of 183 colon adenocarcinomas, point mutations were found in 73% of cases. We obtained the similar conclusion that mutation of *APC* gene may be the important impact of colorectal cancer, as heatmap shown in Figure 4 that the mean number of *APC* gene mutation in colorectal cancer is more than that in other cancers except rectum adenocarcinoma. It can be explained that they are two physiologically proximal cancers. Mutation in *IDH1* gene can reduce cell survival, proliferation and invasion of human glioma (Cui et al., 2016). Mutation in *IDH1* gene is an oncogenic driver in a majority of lower-grade gliomas and have an impact on brain lower grade glioma with different genetic pathway (Ohno et al., 2013; Pieper et al., 2014; Ohka et al., 2017). The same conclusion was acquired in Figure 4 that the mean number of *IDH1* gene mutation in Brain lower grade glioma is more than that in other cancers.

*ACPP* gene plays a vital key in prostate adenocarcinoma (Maatman et al., 1984; Drago et al., 1989; Vihko et al., 2005). From the heatmap, it is clear that the level of *ACPP* gene expression in prostate adenocarcinoma is higher than that in other cancers. The expression levels of TG were found to be altered in all kinds of thyroid carcinomas (Makhlouf et al., 2016). From Figure 3, we obtained similar results that the level of *TG* gene expression in thyroid carcinomas is higher than that in other cancers.

Molecular profiling of tissue-specific genes can be utilized to identify the primary site of tumor. Combination of data of somatic mutation and gene expression profiling were first proposed in this study to predict the primary origin. We obtained considerable prediction performance, and therefore this research can bring great help to the identification of cancer tissue origin. However, we did not carry out research to discover the relationship between data of gene expression and somatic mutation. Our method cannot classify physiologically proximal cancers yet. And it is also a future work to employing other machine learning algorithms that can improve the classification performance.

## Conclusion

Identification of cancer tissue origin is a challenging work recently and in the future. With a lot of molecular profiling available, we can make use of them alone and combine some of them to improve performance of identification primary site of tumor. Machine learning algorithm is also an effective tool to help classifying the cancers. The prediction performance can be tremendously affected by the number of features used.

In this study, we used both molecular data of somatic mutation and gene expression profiling to generate a feature matrix. Then the optimal number of genes was obtained and the data was trained, based on random forest algorithm. The performance of using our method was also compared to only by using data of somatic mutation or gene expression profiling. Our method achieved highest accuracy. Experiment results shows that our method can be an effective tool for primary origin tracing.

## Data Availability Statement

Publicly available datasets were analyzed in this study. This data can be found here: https://dcc.icgc.org/releases/release_26/, https://dcc.icgc.org/releases/release_28/.

## Author Contributions

JY, GT, and PB conceived the concept of the work. BH, XL, BW, and JL performed the experiments. BH and XL wrote the manuscript. QL, WG, and JH reviewed the manuscript. All authors approved the final version of this manuscript.

## Conflict of Interest

BW, JL, XL, QL, GT, and JY were employed by the company Geneis Beijing Co., Ltd. The remaining authors declare that the research was conducted in the absence of any commercial or financial relationships that could be construed as a potential conflict of interest.

## Footnotes

1 https://dcc.icgc.org/releases/release_26/

2 https://dcc.icgc.org/releases/release_28/

## References

[B1] Ambrosini V. Nanni C. Rubello D. Moretti A. Battista G. Castellucci P. (2006). 18F-FDG PET/CT in the assessment of carcinoma of unknown primary origin. *Radiol. Med.* 111 1146–1155. 10.1007/s11547-006-0112-6 17171520

[B2] Baudis M. (2007). Genomic imbalances in 5918 malignant epithelial tumors: an explorative meta-analysis of chromosomal CGH data. *BMC Cancer* 7:226. 10.1186/1471-2407-7-226 18088415 PMC2225423

[B3] Beroukhim R. Getz G. Nghiemphu L. Barretina J. Hsueh T. Linhart D. (2007). Assessing the significance of chromosomal aberrations in cancer: methodology and application to glioma. *Proc. Natl.Acad. Sci. U.S.A.* 104 20007–20012. 10.1073/pnas.0710052104 18077431 PMC2148413

[B4] Birnbaum D. J. Laibe S. Ferrari A. Lagarde A. Fabre A. J. Monges G. (2012). Expression profiles in stage II colon cancer according to APC gene status. *Transl. Oncol.* 5 72–76. 10.1593/tlo.11325 22496922 PMC3323927

[B5] Bloom G. Yang I. V. Boulware D. Kwong K. Y. Coppola D. Eschrich S. (2004). Multi-platform, multi-site, microarray-based human tumor classification. *Am. J. Pathol.* 164 9–16. 10.1016/S0002-9440(10)63090-8 14695313 PMC1602228

[B6] Breiman L. (2001). Random forests. *Mach. Learn.* 45 5–32.

[B7] Centeno B. A. Bloom G. Chen D.-T. Chen Z. Gruidl M. Nasir A. (2010). Hybrid model integrating immunohistochemistry and expression profiling for the classification of carcinomas of unknown primary site. *J. Mol. Diagn.* 12 476–486. 10.2353/jmoldx.2010.090197 20558571 PMC2893632

[B8] Cui D. Ren J. Shi J. Feng L. Wang K. Zeng T. (2016). R132H mutation in IDH1 gene reduces proliferation, cell survival and invasion of human glioma by downregulating Wnt/β-catenin signaling. *Int. J. Biochem. Cell Biol.* 73 72–81. 10.1016/j.biocel.2016.02.007 26860959

[B9] Dietlein F. Eschner W. (2014). Inferring primary tumor sites from mutation spectra: a meta-analysis of histology-specific aberrations in cancer-derived cell lines. *Hum. Mol. Genet.* 23 1527–1537. 10.1093/hmg/ddt539 24163242

[B10] Drago J. R. Badalament R. A. Wientjes M. G. Smith J. J. Nesbitt J. A. York J. P. (1989). Relative value of prostate-specific antigen and prosttic acid phosphatase in diagnosis and management of adenocarcinoma of prostate ohio state university experience. *Urology* 34 187–192. 10.1016/0090-4295(89)90369-5 2477931

[B11] Erlander M. G. Ma X.-J. Kesty N. C. Bao L. Salunga R. Schnabel C. A. (2011). Performance and clinical evaluation of the 92-gene real-time PCR assay for tumor classification. *J. Mol. Diagnost.* 13 493–503. 10.1016/j.jmoldx.2011.04.004 21708287 PMC3157614

[B12] Erlander M. G. Moore M. W. Cotter P. Reyes M. Stahl R. Hamati H. (2004). Molecular classification of carcinoma of unknown primary by gene expression profiling from formalin-fixed paraffin-embedded tissues. *J. Clin. Oncol.* 22:9545. 10.1200/JCO.2007.14.4378 18802157

[B13] Fencl P. Belohlavek O. Skopalova M. Jaruskova M. Kantorova I. Simonova K. (2007). Prognostic and diagnostic accuracy of [18F]FDG-PET/CT in 190 patients with carcinoma of unknown primary. *Eur. J. Nucl. Med. Mol. Imaging* 34 1783–1792. 10.1007/s00259-007-0456-8 17541584

[B14] Fu Z. Chen X. Yang X. Li Q. (2019). Diagnosis of primary clear cell carcinoma of the vagina by 18F-FDG PET/CT. *Clin. Nucl. Med.* 44 493–494. 10.1097/RLU.0000000000002463 30688732

[B15] Greco A. F. (2013). Cancer of unknown primary or unrecognized adnexal skin primary carcinoma? Limitations of gene expression profiling diagnosis. *J. Clin. Oncol.* 31 1479–1481. 10.1200/JCO.2012.47.1615 23439748

[B16] Gross-Goupil M. Massard C. Lesimple T. Merrouche Y. Blot E. Loriot Y. (2012). Identifying the primary site using gene expression profiling in patients with carcinoma of an unknown primary (CUP): a feasibility study from the GEFCAPI. *Onkologie* 35 54–55. 10.1159/000336300 22310348

[B17] Hainsworth J. D. Rubin M. S. Spigel D. R. Boccia R. V. Raby S. Quinn R. (2013). Molecular gene expression profiling to predict the tissue of origin and direct site-specific therapy in patients with carcinoma of unknown primary site: a prospective trial of the sarah cannon research institute. *J. Clin. Oncol.* 31 217–223. 10.1200/JCO.2012.43.3755 23032625

[B18] Hoadley K. A. Yau C. Wolf D. M. Cherniack A. D. Tamborero D. Ng S. (2014). Multiplatform analysis of 12 cancer types reveals molecular classification within and across tissues of origin. *Cell* 158 929–944. 10.1016/j.cell.2014.06.049 25109877 PMC4152462

[B19] Hudis C. A. (2007). Trastuzumab–mechanism of action and use in clinical practice. *N. Engl. J. Medi.* 357 39–51. 10.1056/NEJMra043186 17611206

[B20] Huebner G. Morawietz L. Floore A. Buettner R. Folprecht G. Stork-Sloots L. (2007). Comparative analysis of microarray testing and immunohistochemistry in patients with carcinoma of unknown primary – CUP syndrome. *Eur. J. Cancer Suppl.* 5 90–91.

[B21] Hyphantis T. Papadimitriou I. Petrakis D. Fountzilas G. Repana D. Assimakopoulos K. (2013). Psychiatric manifestations, personality traits and health-related quality of life in cancer of unknown primary site. *PsychoOncol.* 22 2009–2015. 10.1002/pon.3244 23359412

[B22] Janick S. Elodie L.-M. Marie-Christine M. Philippe R. Marius I. (2018). Immunohistochemistry for diagnosis of metastatic carcinomas of unknown primary site. *Cancers* 10:108 10.3390/cancers10040108 PMC5923363 29621151

[B23] Kandalaft P. L. Gown A. M. (2015). Practical applications in immunohistochemistry: carcinomas of unknown primary site. *Arch. Pathol. Lab. Med.* 140 508–523. 10.5858/arpa.2015-0173-CP 26457625

[B24] Kao K. J. Cheng S. H. Huang A. T. (2006). Gene expression profiling for prediction of distant metastasis and survival in primary nasopharyngeal carcinoma. *J. Cli. Oncol.* 24 5503–5503.

[B25] Kuhnen C. Herter P. Monse H. Kahmann S. Muehlberger T. Vogt P. M. (2000). APC and β-catenin in alveolar soft part sarcoma (ASPS) - immunohistochemical and molecular genetic analysis. *Pathol. Res. Pract.* 196 299–304. 10.1016/s0344-0338(00)80059-x 10834386

[B26] Kurahashi I. Fujita Y. Arao T. Kurata T. Koh Y. Sakai K. (2013). A microarray-based gene expression analysis to identify diagnostic biomarkers for unknown primary cancer. *PloS One* 8:e63249. 10.1371/journal.pone.0063249 23671674 PMC3650062

[B27] Küsters-Vandevelde H. V. N. Kruse V. Van Maerken T. Boterberg T. Pfundt R. Creytens D. (2017). Copy number variation analysis and methylome profiling of a GNAQ-mutant primary meningeal melanocytic tumor and its liver metastasis. *Exp. Mol. Pathol.* 102 25–31. 10.1016/j.yexmp.2016.12.006 27974237

[B28] Kwee T. C. Basu S. Cheng G. Alavi A. (2010). FDG PET/CT in carcinoma of unknown primary. *Eur. J. Nucl. Med. Mol. Imaging* 37 635–644. 10.1007/s00259-009-1295-6 19882152 PMC2822231

[B29] Lawrence M. S. Stojanov P. Mermel C. H. Robinson J. T. Garraway L. A. Golub T. R. (2014). Discovery and saturation analysis of cancer genes across 21 tumour types. *Nature* 505 495–501. 10.1038/nature12912 24390350 PMC4048962

[B30] Li Y. Kang K. Krahn J. M. Croutwater N. Lee K. Umbach D. M. (2017). A comprehensive genomic pan-cancer classification using The Cancer Genome Atlas gene expression data. *BMC Genomics* 18:508. 10.1186/s12864-017-3906-0 28673244 PMC5496318

[B31] Ma X. J. Patel R. Wang X. Salunga R. Murage J. Desai R. (2006). Molecular classification of human cancers using a 92-gene real-time quantitative polymerase chain reaction assay. *Arch. Pathol. Lab. Med.* 130 465–473. 10.1043/1543-2165(2006)130[465:MCOHCU]2.0.CO;2 16594740

[B32] Maatman T. J. Gupta M. K. Montie J. E. (1984). The role of serum prostatic acid phosphatase as a tumor marker in men with advanced adenocarcinoma of the prostate. *J. Urolo.* 132 58–60. 10.1016/s0022-5347(17)49463-8 6726962

[B33] Makhlouf A. M. Chitikova Z. Pusztaszeri M. Berczy M. Dibner C. (2016). Identification of CHEK1, SLC26A4, c-KIT, TPO and TG as new biomarkers for human follicular thyroid carcinoma. *Oncotarget* 7 45776–45788. 10.18632/oncotarget.10166 27329729 PMC5216760

[B34] Malhi A. Gao R. (2005). PCA-based feature selection scheme for machine defect classification. *Instrument. Meas. IEEE Trans.* 53 1517–1525.

[B35] Marquard A. M. Birkbak N. J. Thomas C. E. Favero F. Krzystanek M. Lefebvre C. (2016). TumorTracer: a method to identify the tissue of origin from the somatic mutations of a tumor specimen. *BMC Med. Genomics* 8:58. 10.1186/s12920-015-0130-0 26429708 PMC4590711

[B36] McConechy M. K. Ding J. Senz J. Yang W. Melnyk N. Tone A. A. (2014). Ovarian and endometrial endometrioid carcinomas have distinct CTNNB1 and PTEN mutation profiles. *Modern Pathol.* 27 128–134. 10.1038/modpathol.2013.107 PMC3915240 23765252

[B37] Meyer J. G. Liu S. Miller I. J. Coon J. J. Gitter A. (2019). Learning drug function from chemical structure with convolutional neural networks and random forests. *J. Chem. Inform. Model.* 59 4438–4449. 10.1021/acs.jcim.9b00236 31518132 PMC6819987

[B38] Muthukrishnan M. Rohini R. (2016). “LASSO: a feature selection technique in predictive modeling for machine learning,” in *2016 IEEE International Conference on Advances in Computer Applications (ICACA)*, Coimbatore.

[B39] Nota B. (2016). Gogadget: an R Package for interpretation and visualization of go enrichment results. *Mol. Inform.* 36:1600132. 10.1002/minf.201600132 28000438

[B40] Ohka F. Yamamichi A. Kurimoto M. Motomura K. Tanahashi K. Suzuki H. (2017). A novel all-in-one intraoperative genotyping system for IDH1-mutant glioma. *Brain Tumor Pathol.* 34 91–97. 10.1007/s10014-017-0281-0 28353033

[B41] Ohno M. Narita Y. Miyakita Y. Matsushita Y. Shibui S. (2013). Secondary glioblastomas with IDH1/2 mutations have longer glioma history from preceding lower-grade gliomas. *Brain Tumor Pathol.* 30 224–232. 10.1007/s10014-013-0140-6 23494632

[B42] Oien K. A. Dennis J. L. (2012). Diagnostic work-up of carcinoma of unknown primary: from immunohistochemistry to molecular profiling. *Ann. Oncol.* 23 (Suppl._10), x271–x277. 10.1093/annonc/mds357 22987975

[B43] Pieper R. O. Ohba S. Mukherjee J. (2014). Mutant idh1-driven cellular transformation increases rad51-mediated homologous recombination and Temozolomide (Tmz) resistance. *Cancer Res.* 74 4836–4844. 10.1158/0008-5472.CAN-14-0924 25035396 PMC4154998

[B44] Qu K. Z. Li H. Whetstone J. D. Sferruzza A. D. Bender R. A. (2007). Molecular identification of carcinoma of unknown primary (CUP) with gene expression profiling. *J. Clin. Oncol.* 25 21024–21024.

[B45] Rizwan M. Zulfiqar M. (2010). Carcinoma of unknown primary. *J. Pakistan Med. Assoc.* 60 598–599. 20578621

[B46] Rosenwald S. Gilad S. Benjamin S. Lebanony D. Dromi N. Faerman A. (2010). Validation of a microRNA-based qRT-PCR test for accurate identification of tumor tissue origin. *Mod. Pathol.* 23 814–823. 10.1038/modpathol.2010.57 20348879

[B47] Sandri M. Zuccolotto P. (eds) (2006). *Variable Selection Using Random Forests. Data Analysis, Classification and the Forward Search.* Berlin: Springer.

[B48] Shaw P. H. S. Adams R. Jordan C. Crosby T. D. L. (2007). A clinical review of the investigation and management of carcinoma of unknown primary in a single cancer network. *Clin. Oncol.* 19 87–95. 10.1016/j.clon.2006.09.009 17305260

[B49] Sheffield B. S. Tessier-Cloutier B. Li-Chang H. Shen Y. Pleasance E. Kasaian K. (2016). Personalized oncogenomics in the management of gastrointestinal carcinomas-early experiences from a pilot study. *Curr. Oncol.* 23 68–73. 10.3747/co.23.3165 28050146 PMC5176383

[B50] Varadhachary G. R. Raber M. N. Matamoros A. Abbruzzese J. L. (2008). Carcinoma of unknown primary with a colon-cancer profile-changing paradigm and emerging definitions. *Lancet Oncol.* 9 596–599. 10.1016/S1470-2045(08)70151-7 18510991

[B51] Vihko P. T. Quintero I. Rönkä A. E. Herrala A. Jäntti P. Porvari K. (2005). Prostatic acid phosphatase (PAP) is PI(3)P-phosphatase and its inactivation leads to change of cell polarity and invasive prostate cancer. *Cancer Res.* 65 62–78.

[B52] Voigt J. J. (2008). Immunohistochemistry: a major progress in the classification of carcinoma of unknown primary. *Oncologie* 10 693–697.

[B53] Waardenberg A. J. Bassett S. D. Bouveret R. Harvey R. P. (2016). Erratum to: ‘CompGO: an R package for comparing and visualizing Gene Ontology enrichment differences between DNA binding experiments’. *BMC Bioinform.* 17:179 10.1186/s12859-015-0701-2 PMC4845484 27113060

[B54] Ye J. Fang L. Zheng H. Zhang Y. Chen J. Zhang Z. (2006). WEGO: a web tool for plotting GO annotations. *Nucleic Acids Res.* 34 293–312. 10.1093/nar/gky400 16845012 PMC1538768

[B55] Yu G. Wang L.-G. Han Y. He Q.-Y. (2012). clusterProfiler: an R Package for comparing biological themes among gene clusters. *Omics J. Integra. Biol.* 16 284–287. 10.1089/omi.2011.0118 22455463 PMC3339379

